# Financial and Safety Impact of Simulation-based Clinical Systems Testing on Pediatric Trauma Center Transitions

**DOI:** 10.1097/pq9.0000000000000578

**Published:** 2022-08-26

**Authors:** Sacha A. Williams, Katie Fitzpatrick, Nicole M. Chandler, Jennifer L. Arnold, Christopher W. Snyder

**Affiliations:** From the * Division of Pediatric Surgery, Johns Hopkins All Children’s Hospital, St. Petersburg, Fla.; † Department of Surgery, Center for Medical Simulation and Innovative Education, Johns Hopkins All Children’s Hospital, St. Petersburg, Fla.

## Abstract

Simulation offers multiple tools that apply to medical settings, but little is known about the application of simulation to pediatric trauma workflow changes. Our institution recently underwent significant clinical changes in becoming an independent pediatric trauma center. We used a simulation-based clinical systems testing (SbCST) approach to manage change-associated risks. The purpose of this study was to describe our SbCST process, evaluate its impact on patient safety, and estimate financial costs and benefits.

**Methods:**

SbCST consisted of the following steps: (1) change-based needs assessment, in which stakeholders developed relevant simulation scenarios; (2) scenario implementation; and (3) postsimulation failure mode and effects analysis (FMEA) to identify latent safety threats (LSTs). LSTs were prioritized for mitigation based on the expected probability and severity of adverse event occurrences. We calculated the costs associated with the simulation process. We conservatively estimated SbCST cost savings using 3 approaches: (1) FMEA-based avoidance of adverse events; (2) avoidance of trauma readmissions; and (3) avoidance of medical liability lawsuits.

**Results:**

We implemented 2 simulation scenarios prechange. FMEA revealed 49 LSTs, of which 9 were highest priority (catastrophic severity and high likelihood of occurrence). These were prioritized and mitigated using the hospital’s quality/safety framework. Cost-benefit analysis based on FMEA event avoidance demonstrated net cost savings to the institution ranging from $52,000−227,000 over the 3-month postchange period. Readmission-based and liability-based estimates also produced favorable results.

**Conclusions:**

The SbCST approach identified multiple high-impact safety risks and financially benefited the institution in managing significant pediatric trauma clinical process changes.

## INTRODUCTION

Effective clinical workflow processes optimize patient outcomes, enhance regulatory compliance, and improve patient and staff satisfaction.^1,2^ Simulation-based clinical systems testing (SbCST) is one approach for evaluating workflow processes. SbCST uses realistic, in-situ, simulated clinical scenarios to identify flaws and hazards in workflows and systems of care.^3–5^ Importantly, it differs from other simulation approaches that seek to educate or test clinical skills. SbCST does not attempt to teach, but rather to identify latent safety threats (LSTs), defined as medical errors, flaws, and hazards inherent in our clinical environments, processes, and systems of care that result from faulty methods, training, or strategies that impact the well-being of patients and staff.^6–8^

Once LSTs are identified, they can be better characterized and prioritized using failure mode and effects analysis (FMEA), an established tool for investigating failures, their etiology, and their consequences.^9–12^ The utility and benefits of FMEA have been recognized by the Agency for Healthcare Research and Quality, the Institute for Healthcare Improvement, and the Joint Commission. Terminology and definitions are provided for reference in Table 1.

**Table 1.** Terminology and Definitions

Abbreviation	Term	Definition
SbCST	Simulation-based clinical systems testing	Use of realistic, in situ, simulation scenarios to identify weaknesses and hazards in clinical workflows and systems of care.
LST	Latent safety threat	A hazard to patients and staff that is inherent in clinical environments and workflow processes. These hazards result from faulty methods, training, or strategies. They typically become evident only under certain circumstances (ie, when “the holes in the swiss cheese line up”).
FMEA	Failure modes and effects analysis	A tool initially developed by the aerospace industry for investigating failures, their etiologies, and their consequences. The tool has been adapted for healthcare by the Institute for Healthcare Improvement. It quantifies a process failure’s severity and probability of recurrence, allowing prioritization for mitigation.

The utilization of SbCST with its accompanying tools allows a systematic, standardized, multidisciplinary evaluation of clinical workflow processes without putting actual patients at risk.^13–15^ Although the SbCST approach has been applied to multiple areas of medicine, it has not yet found wide application in the field of trauma. Pediatric trauma, a field prone to high-acuity but low-frequency clinical management scenarios, may benefit from an SbCST approach.

Recently, our institution underwent major changes in workflows and processes of care by becoming an independent pediatric trauma facility. Before the transition, injured children were brought from the field to the adjacent adult hospital emergency center (EC). Urgent interventions were performed at the adult hospital, a completely separate entity. After the transition, injured children were brought directly to the children’s hospital EC. Therefore, the major process changes involved managing higher acuity, potentially unstable patients from the field, and performing emergency procedures in the trauma bay or operating room (OR). We used an SbCST approach before the transition to help optimize workflows and ensure patient safety. The aims of this study were (1) to describe the SbCST process in a systematic and reproducible fashion as applied to pediatric trauma, and (2) estimate the financial impact of SbCST on the institution.

## METHODS

### Study Design

**Fig. 1.**
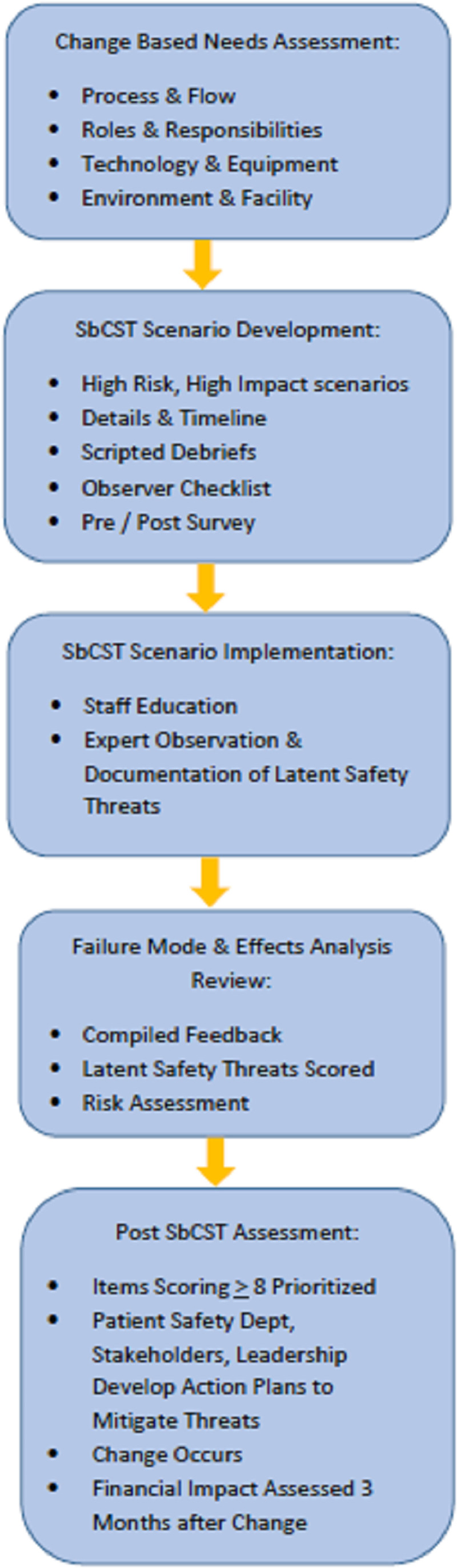
Flowchart of SbCST process.

The study was conducted May–November 2019 at a free-standing tertiary children’s hospital. The study was acknowledged by the institutional review board, but approval was not required. Figure 1 depicts a flowchart of the SbCST process. During the presimulation change-based needs assessment, clinical and administrative stakeholders from multiple departments (trauma/surgery, emergency medicine, OR, nursing, respiratory therapy, etc.) met with simulation center staff to identify potential and anticipated changes in 4 primary areas: (1) patient care workflow; (2) roles and responsibilities; (3) technology and equipment; and (4) environment and design. These findings were incorporated into the development of 2 simulated clinical scenarios for SbCST.

### Simulation Environment

The SbCST was implemented in 2 different settings: (1) during a regular, fully-staffed busy elective OR day and (2) after-hours with minimal staffing. All scenarios were conducted in-situ in the following working clinical environments: helipad, EC, trauma resuscitation areas in the EC, and OR. A high-fidelity mannequin with programmable vital signs monitor was utilized, allowing realistic trauma interventions and actual responses in real time.

### Participants

Implementation of the SbCST included clinical, operational, and administrative participants such as pediatric trauma surgeons, trauma nurses, anesthesia providers, pharmacists, emergency medicine physicians, pediatric intensive care unit (PICU), emergency medical services, simulation, patient transport, and security. The SbCST participant demographics (n = 37) consisted of physicians (22%), nurses (24%), ancillary staff (22%), and other (most indirect patient care, for example, IT support, laboratory manager, etc; 32%). Thirty-eight clinical observers, including the blood bank manager, physicians from the EC, PICU, medical emergency/code team, infectious disease, trauma surgery, anesthesia, or radiology departments, pharmacists, nursing, IT, and emergency medical services, were involved. We briefed participants about general simulation goals and expectations via email. However, the actual time and details of the event were unannounced. Observers underwent a standard presimulation briefing.

### Simulation Scenarios

The first scenario involved a 3-year-old involved in a motor vehicle accident who presented with a femur fracture and hypovolemic shock. This patient required fluid/blood resuscitation in the EC and surgical intervention during peak work hours. The second scenario involved a 7-year-old presenting with a gunshot wound. This patient required emergent transfer to the OR with activation of the massive transfusion protocol, all within the low-resource setting of the night shift. Appendix A, Supplemental Digital Content, which shows full scenario details http://links.lww.com/PQ9/A387, http://links.lww.com/PQ9/A388 [link to online content].

### Debriefing

A 60−90 minute debriefing session immediately followed each SbCST to acquire feedback from participants and observers. This debriefing was led by a trained simulation educator using a scripted debrief guide developed from the initial needs assessment workshop. During these sessions, participants and expert observers reviewed all feedbacks and identified potential LSTs related to resources, environment, process and workflows, and clinical care. Participants and observers were provided with other avenues for unscripted feedback if they could not participate due to work responsibilities.

### Outcomes

FMEA meetings were held approximately 2 weeks after each SbCST and facilitated by a trained simulation educator. At separate FMEA meetings after each SbCST, observer and participant stakeholders, departmental and hospital safety champions, and trauma program leadership reviewed debriefing feedback and each LST using the FMEA toolkit (Table 2). Quality and safety professionals included hospital-employed registered nurses and administrators whose responsibilities included process improvement and root cause analyses for adverse events. Their professional credentials included advanced degrees and extensive experience in patient safety and quality. The severity and probability of occurrence of each LST were scored by consensus with the established FMEA rubric on a Likert scale of 1–4, with severity ranging from minor to catastrophic and probability ranging from remote to frequent. These severity and probability scores were multiplied to assign the LST an overall risk priority number. For example, a threat that was both catastrophic and frequent would receive a risk priority number of 4 × 4 = 16, indicating the highest priority for mitigation. Scoring discrepancies were resolved by further discussion, and consensus was achieved in all cases. Following the FMEA meeting, relevant participants developed action plans to remediate the LSTs, focusing on those with the highest risk priority numbers.

**Table 2.** FMEA Scoring Tool

Score	4	3	2	1
Severity Categories	Catastrophic	Major	Moderate	Minor
Patient outcomes	• Death or major permanent loss of function (sensory, motor, physiologic, or intellectual)	• Permanent lessening of bodily functioning (sensory, motor, physiologic, or intellectual)	• Increased length of stay or increased level of care for 1 or 2 patients	• No injury, nor increased length of stay nor increased level of care
	• Suicide	• Disfigurement		
	• Rape	• Surgical intervention required		
	• Hemolytic transfusion reaction	• Increased length of stay or increased level of care for 3 or more patients		
	• Surgery/procedure on the wrong patient or wrong body part			
	• Infant abduction			
Probability categories	Frequent	Occasional	Uncommon	Remote
	Likely to occur immediately or within a short period (may happen several times in 1 y)	Probably will occur (may happen several times in 1–2 y)	Possible to occur (may happen sometime in 2–5 y)	Unlikely to occur (may happen sometime in 5–30 y)
Equipment/facility damage	>$250,000	$100,000–$250,000	$10,000–$100,000	<$10,000, or loss of utility

RPN is calculated by multiplying severity score (1−4) by probability score (1−4). Issues are considered significant priorities if RPN is between 8 and 16 on a scale of 1−16.

RPN, risk priority number.

Adapted with permission from Institute for Healthcare Improvement.^9^

We analyzed financial costs and benefits associated with SbCST. Personnel costs were calculated by identifying each participant and the amount of their time required to participate in the SbCST. Subsequently, each employee’s time was multiplied by their hourly pay rate. Equipment, facility, and other miscellaneous resource costs were provided by the simulation center. Mitigation strategies for LSTs were developed outside the simulation environment by the relevant clinical departments, in conjunction with the hospital quality and safety department. Mitigation strategy development and implementation were not included in the cost model because these strategies fell within the hospital’s existing fixed costs.

There is no universally accepted method for estimating financial benefits of SbCST in introducing a new process.^16,17^ We attempted to follow the 6-point approach proposed by Schmidek and Weeks^18^ for evaluating financial return on investment related to patient safety initiatives. A key element of this approach is addressing uncertainties by using multiple outcomes and varying assumptions (ie, sensitivity analyses). We modeled validated outcomes, covering several related domains: patient safety, clinical, and medicolegal.^19^ For each outcome method, a conservative range of assumptions was used to allow for robust sensitivity analysis.

The first method used the FMEA described previously. The FMEA rubric is an established tool in the patient safety/quality space, and its successful integration into SbCST has been previously described.^20^ It was also the tool with which the authors had the most experience. Each LST scored in the FMEA had an expected frequency of adverse event occurrence, and a range of expected costs the institution would be expected to bear each time the associated adverse event occurred.^13^ Similar individual LSTs were grouped together as much as possible to eliminate redundancy and create a parsimonious list of LSTs. For each of these LSTs, the number of events expected to occur during the first 3 months after the new process go-live date was determined based on the expected baseline rate and the expected percent reduction in event rate. The expected reduction is not only from a general heightened awareness, but also from involving the hospital’s existing quality/safety processes to target the threats. LSTs identified with SbCST were treated as “real-world” near-misses or actual patient harm events. Previous studies have shown simulation-related reductions in adverse event rates of up to 100%.^21^ Expected percent reduction was conservatively estimated at 25%. An even more conservative 10% reduction was also evaluated. The minimum expected cost associated with each event occurrence was multiplied by the expected number of events avoided. The expected cost savings associated with adverse event avoidance during this 3-month time period were added together to estimate a total financial benefit.

The second method approximated cost savings based on avoidance of trauma readmissions. The degree to which readmissions result in direct incremental costs to the institution is difficult to quantify, but they are likely to produce disproportionate financial losses for children’s hospitals.^22–25^ Unplanned hospital readmissions occur after 5%−25% of admissions for trauma and are associated with in-hospital adverse events.^26–28^ Simulation-based quality improvement initiatives are associated with reductions in readmissions from 12% to 50% below the baseline readmission rate.^29,30^ Our calculation was based on the following assumptions derived from historical data: 450 trauma admissions/y with a 10% unplanned readmission rate. Reductions in readmissions were conservatively estimated between 15% and 25%. Cost per readmission after injury was derived from published data.^31^ Cost savings over the 3-month period were calculated based on 15% and 25% reduction estimates.

The third method estimated cost savings by avoidance of medical malpractice claims. The cost savings associated with circumventing a single medical malpractice suit were calculated based on previous published studies of average costs per malpractice claim, relationships of adverse events to claims, and simulation-related reductions in claims.^32–37^ The costs and savings associated with the SbCSTs were normalized to 2019 US dollars using the US Department of Labor calculator.^38^ The 3 estimation methods described were compared in tabular form.

## RESULTS

The SbCSTs yielded 49 LSTs, 38 of which scored 8 or above (high priority) on the FMEA scale. The LSTs were grouped into 4 categories: systems (n = 29, 59%), clinical performance (n = 11, 22%), resources (n = 5, 10%), and facilities (n = 4, 8%). Nine LSTs scored 16, indicating highest severity and most frequent probability of occurrence. These highest-priority LSTs and their mitigation strategies are summarized in Table 3.

**Table 3.** Highest Priority* LSTs Identified during Simulation-based Clinical Systems Tests

Threat Type	Details	Threat Mitigation Strategies
Resource issues (issues related to personnel, medication, and equipment—whether missing, malfunctioning, or unable to use due to lack of provider familiarity with the device)		
Equipment/supply availability	Ran out of essential medications (calcium) in OR. No one available to go to pharmacy to replenish.	• Overstock trauma rooms to have a surplus of essential medications.
		• Trauma medication box containing additional emergency medications prepositioned in OR core and in ER.
Staffing	Delay in OR room setup/availability. Overnight scrub tech had called out sick, requiring the OR nurse to simultaneously set up room, call staff in, and answer phone calls.	• New staffing policy—if someone calls out, backup RN required to come in-house to be immediately available to help (previously home call).
		• Designate a specific OR as the overnight trauma room and presetup as much as possible without wasting supplies.
Systems issues (issues related to process, policies, or procedures that do not work as well as anticipated in the clinical setting)		
Communications-anesthesia	Anesthesia did not get Rave Alert for level 1 trauma activation. Did not respond—had to be notified by phone call from Trauma Surgeon.	• Anesthesiologist/CRNA on call to carry trauma pager—Rave text messages can be carrier-dependent and therefore delayed.
Communications-OR	OR nurse not notified that patient was coming up emergently to OR—unprepared for patient. Phone call notification was attempted but phone busy.	• OR front desk staff member/on-call RN to carry trauma pager at all times
		• OR RN to come down to trauma bay for Level 1 activations if available—facilitate communication with trauma team leader and anticipation of OR needs
Massive transfusion protocol	Despite protocol activation, trauma team had to call blood bank multiple times for platelets.	• Platelets should be automatically dispensed with every other pack of products
		• Review of protocol and reeducation of blood bank staff
System access	OR staff locked out of Pyxis system (automated supply storage cabinet) on emergent case	• Override set up so that Pyxis automatically remains open on emergent case
		• Manual override key available at the OR front desk as backup—staff education
Facility issues (facility or space set up concerns that are not conducive to effective, efficient, and safe patient care)		
Communication system	Existing communication systems (Vocera, text message) can be unreliable/delayed in OR. No overhead paging system in the OR.	• Facility in process of installing equipment to boost signal
		• No good communication system alternatives identified at this time
Clinical performance issues (related to cognitive skills, technical skills, or institutional process knowledge of clinical personnel that can be a focus for future simulation-based training)		
Role delineation	Lack of role clarity—difficult for staff/documenter to know who was in the room. No time for initial introductions due to immediate patient arrival and staggered arrival of other responders.	• Identify roles upon arrival/prebrief if time allows
		• Use role stickers—hanging outside of trauma room, to be quickly stuck outside cap or gown for easy visibility
Crowd control/noise level/closed-loop communication	Documenter unable to hear what interventions and medications were given. Pharmacy was asked multiple times for rapid sequence intubation medications, could not hear attending.	• List of essential personnel (required to be in room) posted outside trauma bay. Others wait outside until called in by trauma team leader.
		• Designated person posted outside trauma bay for crowd control
		• Rapid-cycle deliberate-practice training for clear, closed-loop communication/repeat back

*Defined as highest severity (catastrophic) and highest probability of occurrence (frequent) from FMEA; risk priority number 4 × 4 = 16.

ER, emergency room; RN, registered nurse; CRNA, Certified Registered Nurse Anesthetist.

The analysis of the financial impact of SbCSTs is presented in Table 4. All FMEA-based methods showed cost savings, with a minimum of $52,138 and maximum of $227,138 saved, depending on the assumptions used. The readmission-based methods showed a range of financial impacts depending on the assumptions used, from 3-month costs of $976 under the most conservative assumptions to 3-month savings of $9967 under less conservative assumptions. Medicolegal liability-based methods showed substantial cost savings when at least 1 event was avoided.

**Table 4.** Estimated Costs versus Savings Related to Simulation-based Clinical Systems Tests for Initial 3-month Period after New Process Adoption

Costs or Savings Item	Amount, 2019 US Dollars	Savings Minus Costs (Estimated Net Savings), 2019 US Dollars
Costs		
Personnel: planning	$2493	—
Personnel: implementation	$11,984	—
Personnel: debriefing	$2482	—
Personnel: FMEA	$2482	—
Simulation center resources—day 1	$1921	—
Simulation center resources—day 2	$1500	—
Total costs	$22,862	—
Savings		
FMEA-based		
10% risk reduction, all highest-priority LSTs (n = 8)*	$100,000	$77,138
25% risk reduction, all highest-priority LSTs (n = 8)	$250,000	$227,138
10% risk reduction, resource/system LSTs only (n = 6)	$75,000	$52,138
25% risk reduction, resource/system LSTs only (n = 6)	$187,500	$164,638
Readmission-based		
15% risk reduction (avoidance of 2 readmits over 3 months)	$21,886	($–976)
25% risk reduction (avoidance of 3 readmits over 3 months)	$32,829	$9,967
Medical malpractice liability-based		
Avoidance of 1 event	$135,994	$113,132
Avoidance of 2 events	$265,613	$242,751

*See Table 2 for full list of highest priority LSTs.

## DISCUSSION

This study describes a systematic utilization of simulation tools to identify and mitigate potential threats to trauma patient safety in the setting of major pediatric trauma center workflow changes. Rather than identifying LSTs reactively via morbidity/mortality conferences or root cause analyses of adverse patient events, SbCST allowed a proactive approach. Our study builds on previous work showing a framework by which SbCST and associated tools can be combined systematically and integrated with existing hospital quality and safety initiatives. This study is novel in its practical application of SbCST to pediatric trauma workflow. Another unique aspect of this study is the demonstration of favorable cost-benefit calculus for the SbCST approach in managing pediatric trauma workflow changes. With increasing economic constraints, trauma centers may find their simulation resources threatened. Studies such as ours help communicate the “bottom line” financial benefits of simulation and justify maintaining these important resources.

This comprehensive SbCST approach was in line with previous work by Colman et al,^14,15^ Nielsen et al,^13^ Adler et al,^39^ and Geis et al,^40^ who employed these techniques in their evaluation of LSTs arising from the development of novel healthcare institutions. This methodology has yet to gain broad traction in the healthcare arena, as these tools are often deemed too complex, time-consuming, and/or expensive. Various components of simulation have been applied successfully to patient safety,^4^ operational readiness,^41^ multidisciplinary teamwork,^42^ and skills training in critical scenarios such as adult trauma,^43^ pediatric emergency departments,^44^ and rapid responses.^45^ FMEA has been used in the evaluation of radiation and chemotherapy implementation,^10^ and communication during transplantation.^6^ However, given the bewildering array of simulation tools and approaches, systematically linking appropriate tools into a comprehensive whole can be challenging.

We did find that the SbCST approach required a significant commitment of time and human effort. Planning required consideration of communications, reports, and human and facility resources. The multidisciplinary nature of the SbCST scenarios meant that their implementation needed extensive coordination among clinical departments. Simulation center staff led the planning, implementing, and debriefing phases with extensive participation from front-line clinicians. The resultant LSTs highlighted issues in resources, systems, facilities, and clinical performance. Most were high priority, requiring immediate attention and improvement to prevent miscommunication, delayed care, staff dissatisfaction, and/or patient harm. Moreover, since greater than half were systems issues surrounding notifications and delays, the majority were straightforward and easy to rectify. We found that having hospital patient safety officers and departmental safety champions participating in the FMEA was important because potentially catastrophic LSTs “got their attention.” The hospital’s quality and safety personnel could then take an active role in implementing action plans. The specifics of each action plan depended on the nature of the LST. For example, the issue involving the OR staff being locked out of the Pyxis could be solved by a simple process change (setting up automatic override for trauma cases) and staff education regarding the location of the physical override key. The OR nursing staff problem required more complex nursing leadership intervention. This did not involve hiring new staff, but rather modifying policies to better utilize existing staff. In summary, we found that SbCST brought attention to LSTs, allowing them to be prioritized and fixed within the hospital’s existing quality and safety framework.

We learned specific lessons from our application of SbCST to pediatric trauma workflow. First, simulations must be performed in the actual trauma patient care environment and unannounced (as for real trauma alerts) to achieve the goal of SbCST. The risk of in situ simulation disrupting real-world patient care can be overcome with careful planning and communication. Second, front-line trauma clinicians must be involved in all phases of the SbCST to keep the process anchored to clinical realities. However, simulation workflows can involve lengthy prebriefs and debriefs that may not be feasible for busy front-line clinicians. Simulation personnel may need to modify workflows to facilitate clinician participation. Third, scenario observers should be minimized, to avoid hampering each other’s observations and the participants’ performances. However, hospital quality/safety personnel should observe the scenarios firsthand if possible.

Since there is a paucity of published literature on the cost-effectiveness of trauma simulation to identify and mitigate patient safety risks, comparative studies and established analysis methods were lacking.^46^ Nevertheless, the described financial analysis in this study demonstrated that the SbCST was cost-effective, resulting in substantial cost savings by all estimation methods and assumptions except one. Even that outlier, which assumed a 15% reduction in readmissions and no other benefits, could be considered essentially budget neutral, resulting in net costs of less than $1000 over 3 months. The FMEA-based cost analysis method stemmed from an established, validated quality improvement tool and estimated cost savings to the institution of at least $52,000. These findings are consistent with previous studies of simulation-based training that have shown substantial cost savings.^47,48^

Our findings must be interpreted in the context of some limitations. All in-situ simulations suffer from some artifacts of the controlled environment and may not accurately capture clinical workflows. Only 2 simulation scenarios were used to test specific key workflow changes; they could not comprehensively test all changes. Observation bias and recall bias may have affected the observers and participants. However, we found that time constraints of patient care did not allow busy clinicians to participate in a prolonged combination debriefing/FMEA scoring meeting after each simulation. The time interval between event and FMEA allowed adequate time for compilation and organization of feedback. The consensus-based nature of FMEA may make it prone to cognitive biases. The generalizability of this study to other centers is unclear, as clinical processes and simulation resources vary by institution. However, all hospital systems, and trauma centers, in particular, share many common features that would be expected to make this framework broadly applicable. Due to the lack of validated precedent for financial analysis in this setting, the cost-benefit analysis was mostly theoretical. However, this lack of precedent also highlights the need for novel studies that address simulation cost-benefit questions. All approaches yielded qualitatively similar results.

In conclusion, SbCST was a successful, cost-effective tool for managing significant clinical process changes in transitioning to an independent pediatric trauma center. By describing our process and its financial costs and benefits in detail, we hope that other trauma centers will make use of these valuable tools and better communicate the value of simulation to their leadership teams. Future studies should explore ways to optimize simulation tools for trauma and integrate them into the fabric of facility operations.

## DISCLOSURE

The authors have no financial interest to declare in relation to the content of this article.

## Supplementary Material


