# Supplementary material for: Financial and Safety Impact of Simulation-based Clinical Systems Testing on Pediatric Trauma Center Transitions
Source: Pediatr Qual Saf. 2022 Aug 26;7(5):e578. doi: 10.1097/pq9.0000000000000578 (PMC9416763; doi:10.1097/pq9.0000000000000578)
Supplement: Supplementary file 1 [file pqs-7-e578-s001.pdf]

Simulation Program

Simulation Scenario- SbCST for ____Trauma Protocol__________

Scenario Overview

____

Objectives of Scenario: Using simulation-based clinical system testing methodology, evaluate current planned ____

Specific Testing Priorities based on degree of change, risk, and/impact:

Facilities and Environment:
1. The Trauma Room and the OR was identified in a timely fashion.

Technology and Devices:
1. Appropriate technology is available and accessible in each location.

Processes of Care/Workflows:
1. Notification system is in place and notifies team in a realistic time frame that allows them to prepare for the trauma.
2. Team was mobilized in quickly and there was no delay in care.

Roles and Responsibilities:
1. It is clear what roles are responsible are delineated in the communication algorithm.

Patient Description:

Patient History (Medical, Surgical, Social)
Current MVC rollover, child not properly restrained), contusions over chest/abdomen, abdominal distension, obvious femur fracture with intact pulses, and hypertension Driver of vehicle was pronounced dead at the scene. Airway is intact and the GCS is 14. Patient 18 months 15 kg.

Baseline Vital Signs

HR 170
RR 55
BP 60/40
SaO2 80
Weight 15 kg

Target Participants and Responsibilities:

To involve all appropriate teams in the triage and implementation of care of a level 1 trauma patient

Anticipated Duration:

Scenario Time 1 hour

Debriefing Time (typically 2-3x scenario length)

Scenario Set-UP

<table>
<tr><td>Location and Setting</td><td colspan="4">Room: Insitu</td><td colspan="3">Setting: Interventional Radiology</td></tr>
<tr><td rowspan="2">Mannequin Set Up</td><td>Mannequin</td><td>Wardrobe</td><td>Monitors</td><td>Moulage</td><td>Access</td><td>ID Band Info:</td><td>Other Details</td></tr>
<tr><td>Sim Baby</td><td></td><td></td><td>Bloody Leg, Bruising on abdomin</td><td></td><td></td><td>Tibia fracture</td></tr>
<tr><td>SPs</td><td colspan="7">Character Names and Roles: (Parent)
N/A</td></tr>
<tr><td>Embedded Person Roles</td><td colspan="3">Role (RN, MD, Parent, etc)
1. N/A</td><td colspan="4">Scripts or Hand-Off information:
1. Script for EMS Com Center</td></tr>
<tr><td>Room Staging (environment) and Equipment in Room</td><td colspan="7">☐ Patient Starts off at an outside location</td></tr>
<tr><td>Medical Chart Information</td><td colspan="7">☐ Electronic/Paper Chart required:
☐ Lab Results:
☐ Diagnostic Imaging:</td></tr>
<tr><td>Pre-Sim Checklist</td><td colspan="7">☐ Video recording is enabled
☐ Debriefing location is identified
Vital signs: weight: kg
Heart rate: 170
Blood pressure: 60/40
Respiratory rate:55
Oxygen saturation: 80
Temperature:</td></tr>
</table>

**Expected Participants**	☐ EMS, EC Trauma Team, OR Team, MET Team

Room For Running SbCST: Will be determined when call is received.

Actual/Real vs. Simulated Medical Equipment and Supplies to be set up in space for SbCST include: Real Medicine/Simulated for controlled substances or meds that are constrained

▪

Simulation Equipment Needed:

- Audio recorders
- Camera and video recording set up
- Method for taking notes
- Observer signs for wearing
- Sim Team signs for wearing
- Note cards with patient examination and lab findings as needed

Mannequins/ Task trainers/ Standardized Patients Needed:

Patient Medical Chart Information: Patient information will be entered into Cerner. Labs and Pharmacy orders will be entered in there as well.

ITTESTING, EMERGENCY
MR# 1034334

Demonstration Items needed for Debriefing:

Flip charts for note taking or laptop computer
Scripted debriefings
Observer checklists

Scenario Logistics

Expected Scenario Flow (Flow Chart):

- Participants given pre-briefing:
  - ➢ *The goal of this simulation is to evaluate if we have the optimal processes of care, resources (people, equipment/supplies, etc), layout and space, and necessary skill sets and competencies to care for trauma patients. After we complete this scenario we will debrief to identify and address as many latent safety threats, issues and concerns as possible.*
  - ➢ Current MVC rollover, child not properly restrained), contusions over chest/abdomen, abdominal distension, obvious femur fracture with intact pulses,

and hypertension Driver of vehicle was pronounced dead at the scene. Airway is intact and the GCS is 14. Patient 18 months 15 kg.

Helipad	EC	OR
**State 1:Initial**<br><br>**Expected Interventions:**<br>• Someone from the ER will be at the door to receive patient<br><br>• Helicopter Team/EC rep walk patient Down to the EC<br><br>**Vital Signs**<br>Patient is Stable<br><br>HR 170<br>BP 60/40<br>SATs 100<br>RR (Bagged)	**State 1:Pre Arrival**<br><br>**Expected Interventions:**<br><br>• Level Patient<br>• HUC send out Rave alert/<br>• Preregisters Patient<br>• Pre arrival huddle with Team<br>• Secondary Nurse will call blood bank to confirm level one trauma/need for blood cooler<br>• Tech Will Run to Blood Bank Grab Cooler<br>• Medic will grab warmed fluids (push pull) and surgical equipment<br>• Anesthesia arrives<br>• Trauma Service Practitioner Control Desk calls the OR Team.<br>• Secondary Nurse will call and get med rec number for OR to pull equipment<br>• Radiology Waiting at Bedside<br>• EC staff have PPE and Led	**State 1: Pre Arrival**<br><br>**Expected Interventions:**<br><br>• After Control Desks calls with MRN Pulls Supplies (trauma cart, trauma instruments/supplies.<br>• EC will transport patient up to control desk; OR team will meet EC at desk to take patient<br>• Patient Transferred to OR Table<br>• Patient placed on the monitor<br><br>**Vital Sign**<br><br>**Stable**<br><br>**Vital Signs:**<br>HR 170<br>BP 60/40<br>Sats 100-86<br>RR bagged
**State 2:Transport**	**State 2: Arrival**<br><br>**Pneumo**	**State 2: Arrival/End of Scenario**

**Patient Remains Stable**<br><br>**Expected Interventions:**<br><br>**Vital Signs**<br>HR 170<br>BP 60/40<br>SATs 100<br>RR (bagged)	Patient starts to decompensates (pneumothorax):<br><br>**Expected Intervention:**<br>• RSI<br>• Needle Decompress the Patient<br>• Place Chest tube<br><br>**Vital Signs:**<br>HR 170<br>BP 60/40<br>Sats 88 – 98 (after decompression)<br>RR bagged<br><br>Decreased breath sounds on the left	**Expected Intervention:**<br><br>• Patient Transferred to OR Table<br>• Patient placed on the monitor<br><br>**Vital Signs: Stable**<br>HR 170<br>BP 60/40<br>Sats 100<br>On Anesthesia Machine
	**State 3: Ongoing Tachycardia/Hypotension**<br><br>• Fast Exam completed<br>• Positive for Hemoperitoneum<br>• MTP activation<br>• Emergent transfer to OR<br><br>**Vital Signs:**<br>HR 170<br>BP 50/40<br>Sats 100<br>RR bagged	

Expected Endpoint of the Scenario: Scenario will end once patient is transferred to OR and then placed on OR Table.

Debriefing Points: audio record debriefing

See scripted debriefing
