# Supplementary material for: Financial and Safety Impact of Simulation-based Clinical Systems Testing on Pediatric Trauma Center Transitions
Source: Pediatr Qual Saf. 2022 Aug 26;7(5):e578. doi: 10.1097/pq9.0000000000000578 (PMC9416763; doi:10.1097/pq9.0000000000000578)
Supplement: Supplementary file 2 [file pqs-7-e578-s002.pdf]

Simulation Scenario- SbCST for ____Trauma Protocol__________

Scenario Overview

____

Objectives of Scenario: Using simulation-based clinical system testing methodology, evaluate current planned ______________________________________

Specific Testing Priorities based on degree of change, risk, and/impact:

Facilities and Environment:
1. The Trauma Room and the OR was identified in a timely fashion.

Technology and Devices:
1. Appropriate technology is available and accessible in each location.

Processes of Care/Workflows:
1. Notification system is in place and notifies team in a realistic time frame that allows them to prepare for the trauma.
2. Team was mobilized in quickly and there was no delay in care.

Roles and Responsibilities:
1. It is clear what roles are responsible are delineated in the communication algorithm.

Patient Description:

Patient History (Medical, Surgical, Social)
7 year old male gunshot wound right upper quadrant abdomen. Intubated in field for combativeness. 1 large-bore PIVs placed and IO.
Stable en route, but rapidly deteriorates to hypotensive and tachycardic.

Baseline Vital Signs

HR 130
RR Bagged
BP 65/35
SaO2 92
Weight 20 kg

Target Participants and Responsibilities:

To involve all appropriate teams in the triage and implementation of care of a level 1 trauma patient

Anticipated Duration:

Scenario Time 1 hour

Debriefing Time (typically 2-3x scenario length)

Scenario Set-UP

**Location and Setting**	**Room: Insitu** / **Setting: EC/OR**
**Mannequin Set Up**	(see table below)
**SPs**	Character Names and Roles: (Parent)<br>N/A
**Embedded Person Roles**	**Role (RN, MD, Parent, etc)**: 1. N/A<br>**Scripts or Hand-Off information:** 1. *Script for EMS Com Center*
**Room Staging (environment) and Equipment in Room**	☐ Patient Starts off at an outside location
**Medical Chart Information**	☐ Electronic/Paper Chart required:<br>☐ Lab Results: See last page<br>☐ Diagnostic Imaging:
**Pre-Sim Checklist**	☐ Video recording is enabled<br>☐ Debriefing location is identified<br>Vital signs: weight: kg<br>Heart rate: 130

Mannequin Set Up

Mannequin	Wardrobe	Monitors	Moulage	Access	ID Band Info:	Other Details
Pediatric Hal and Trauma Kid+Surgical Trainer			Gunshot Wound Left Upper Quadrant (Mehron Coagulated Blood) Gunshot Would right abdomen.	R PIV and IO right Leg		Intubated

	Blood pressure: 65/35<br>Respiratory rate:55<br>Oxygen saturation: 92<br>Temperature:
**Expected Participants**	☐ EMS, EC Trauma Team, OR Team, MET Team

Room For Running SbCST: Will be determined when call is received.

Actual/Real vs. Simulated Medical Equipment and Supplies to be set up in space for SbCST include: Real Medicine/Simulated for controlled substances or meds that are constrained

▪

Simulation Equipment Needed:

- Audio recorders
- Camera and video recording set up
- Method for taking notes
- Observer signs for wearing
- Sim Team signs for wearing
- Note cards with patient examination and lab findings as needed

Mannequins/ Task trainers/ Standardized Patients Needed:

Patient Medical Chart Information: Patient information will be entered into Cerner. Labs and Pharmacy orders will be entered in there as well.

ITTESTING, EMERGENCY
MR# 1034334

Demonstration Items needed for Debriefing:

Flip charts for note taking or laptop computer
Scripted debriefings
Observer checklists

Scenario Logistics

Expected Scenario Flow (Flow Chart):

- Participants given pre-briefing:
  - ➢ *The goal of this simulation is to evaluate if we have the optimal processes of care, resources (people, equipment/supplies, etc), layout and space, and necessary skill sets and competencies to care for trauma patients. After we*

complete this scenario we will debrief to identify and address as many latent safety threats, issues and concerns as possible.

**Patient Background**	Mechanism: 7 year old male gunshot wound right upper quadrant abdomen and . Intubated in field for combativeness. 1 large-bore PIVs placed and IO. Stable en route, but rapidly deteriorates to hypotensive and tachycardic.		
**Location**	**Ambulance**	**EC**	**OR**
**State:1**	**Pre Arrival**	**Pre Arrival**	**Pre Arrival**
**Expected Interventions**	• Access Obtained<br>• Patient intubated	• Level Patient<br>• HUC send out Rave alert/<br>• Preregisters Patient<br>• Pre arrival huddle with Team<br>• Secondary Nurse will call blood bank to confirm level one trauma/need for blood cooler<br>• Tech Will Run to Blood Bank Grab Cooler<br>• Medic will grab warmed fluids (push pull) and surgical equipment<br>• Anesthesia arrives<br>• Trauma Service Practitioner Control Desk calls the OR Team.<br>• Secondary Nurse will call and get med rec number for OR to pull equipment<br>• Radiology Waiting at Bedside<br>• EC staff have PPE and Led	• After Control Desks calls with MRN Pulls Supplies (trauma cart, trauma instruments/supplies.<br>• EC will transport patient up to control desk; OR team will meet EC at desk to take patient<br>• Patient Transferred to OR Table<br>• Patient placed on the monitor
**Vitals**	HR 130<br>BP 65/35<br>SATs 92<br>RR Bagged (RR 20 on monitor)	HR 140<br>BP 60/30<br>SATs 89<br>RR 20 on monitor<br><br>Decreased breath Sounds on the Left	HR 160<br>BP 60/30<br>SATs 92-88 (increase after chest tube is placed and then decompensates again due to hemodynamic instability)

	**Patient Arrives in EC**		RR Bagged
**State 2:**	**Arrival in the EC**	**Arrival in Trauma Bay**	**Arrival in the OR**
**Expected Interventions**		• Start Primary assessment<br>• Identify decreased breath sounds	• Place 20 gauge IV or Central<br>• Place A Line<br>• Switch out to Level 1<br>• Begin Meds (pharmacy)<br>• MTP should continue<br>• Place on monitors<br>• Prep and Drape<br><br>(additional mannequin setup – Art Line<br>Central Line)<br>Pause and Switch To SurgiSam after Patient is moved
**Vitals**	HR<br>BP<br>Sats<br>RR	HR 150<br>BP 60/30<br>SATs 89<br>RR Bagged (RR 20 on monitor)<br><br>Decreased breath Sounds on the Left	HR 160<br>BP 50/30<br>Sats 87<br>RR Bagging
**State 3**		**State 3: Ongoing Tachycardia/Hypotension**	
**Expected Interventions**		• Set up atrium<br>• Place chest tube<br>• Give Fluid and Blood<br>• Fast Scan<br>• Activate MTP<br>• May get to Secondary or Transfer to OR	• Surgeon Makes Incision<br>• Find Bleeds<br>• MTP Continues<br>• Cauterizes Bleed/Clamps Bleed<br>• Scenario Ends

**Vitals**	HR<br>BP<br>Sats<br>RR	HR 170<br>BP 60/30<br>SATs 92-90 (increase after chest tube is placed and then decompensates again due to hemodynamic instability)<br>RR Bagged (RR 20 on monitor)	HR 75<br>BP 45/25<br>Sats 82<br>RR - Anesthesia

Expected Endpoint of the Scenario: Scenario will end after MTP is initiated in OR.

Debriefing Points: audio record debriefing

See scripted debriefing

Labs:

Venous Blood Gas
- pH 7.19
- pCO2 56
- pO2 60
- HCO3 20.2
- Base Excess -7.7
- O2 saturation 86
- Lactate poc 4.0
- Hgb 5.8
- Hct POC 18

Chemistry
- BUN 9
- Sodium 137
- Potassium 3.6
- Chloride 108
- CO2 21
- Calcium 7.9
- Glucose 217
- Creatinine 0.35
- Total bili 1.0
- Albumin 2.7
- Total protein 5.1
- ALT 98
- AST 215
- Amylase 90
- Lipase 49

Coags
- PT 15.8
- INR 1.22
- APTT 23.8

Hematology
- WBC 10.7
- HgB 5.5
- Hct 17
- Plt 136
